# Design of a superluminal ring laser gyroscope using multilayer optical coatings with huge group delay

**DOI:** 10.1038/srep07098

**Published:** 2014-11-18

**Authors:** Tianliang Qu, Kaiyong Yang, Xiang Han, Suyong Wu, Yun Huang, Hui Luo

**Affiliations:** 1 College of Optoelectric Science and Engineering, National University of Defense Technology, Changsha 410073, China

## Abstract

We propose and analyze a superluminal ring laser gyroscope (RLG) using multilayer optical coatings with huge group delay (GD). This GD assisted superluminal RLG can measure the absolute rotation with a giant sensitivity-enhancement factor of ~10^3^; while, the broadband FWHM of the enhancement factor can reach 20 MHz. This superluminal RLG is based on a traditional RLG with minimal re-engineering, and beneficial for miniaturization according to theoretical calculation. The idea of using GD coatings as a fast-light medium will shed lights on the design and application of fast-light sensors.

Controlling the speed at which light propagates has been the focus of numerous studies during the past two decades^1^,^2^,^3^. In addition to its fundamental importance, the tunability of light speed also opens up new avenues for diverse applications ranging from optical data buffering to enhanced precision in interferometry. Recently, it has been shown that a fast-light medium can be used to realize an absolute rotation sensor whose sensitivity is enhanced with an enhancement factor as high as 10^6^ 4. In this case, the fast-light enhanced gyroscope might be able to detect the gravitational frame-dragging effect terrestrially via measuring the Lense-Thirring rotation^4^,^5^. This enhancement is induced by a frequency dependent phase shift within a Ring Laser Gyroscope (RLG), which has been shown both theoretically^5^,^6^ and experimentally^7^,^8^ by means of increased “mode pushing” or “mode pulling” effects. Various systems have been investigated in order to achieve optimal performances for superluminal gyroscope applications, including alkali metal vapor cells^7^,^8^, coupled optical resonators^9^, photorefractive crystals^10^, optical fibers (dual-pumped Brillouin gain in a fiber or fiber-coupled whispering gallery resonators)^11^, spectral hole burning^12^, and rare atomic gases^13^.

Alkali metal vapor cells have been widely investigated as anomalous dispersion system. However, additional components are requested facing significant engineering challenges, which is disadvantageous for precise measurement. Moreover, since the operating wavelength is confined by the alkali metal atoms, the applications for alkali metal vapor cells are limited. For example, it is unable to realize a White-Light-Cavity that operates at 1064 nm in the application of a LIGO-like gravitational wave detector^10^. Coupled optical resonators^9^,^14^ can be conveniently employed to provide critical anomalous dispersion within a narrow-band spectral range with the assistance of some passive elements. However, the FWHM bandwidth for the sensitivity enhancement factor is narrow, and the gyro is often too complex with a coupled cavity to accurately control its cavity length^14^. The photonic structures such as photorefractive crystals and optical fibers are more suitable to integrate with practical systems, but they all suffer from fundamental limitations to provide long delays of short pulses^15^,^16^,^17^. The nonlinear effect via spectral hole burning has been employed to induce dispersion which results in a scale factor enhancement of only 0.33~2, and an additional laser is needed^12^. In general, existing anomalous dispersion systems are still far from practical applications. Additional components such as a vapor cell, a pumping laser or a coupled cavity are needed, which makes the gyroscope complex and unstable, leading to additional errors such as backscattering. Therefore, to reach the full potential of fast-light enhanced RLG, novel materials or systems for fast-light are needed which are compact and request minimal re-engineering; the FWHM bandwidth of the enhancement factor should be large (broadband); and the novel materials or systems for fast light should not induce additional backscattering.

Multilayer optical coatings are most effective systems to modulate both the amplitude and phase of light^18^ by means of inducing group delay (GD) and group delay dispersion (GDD). For phase modulation, compared with other dispersive medium, multilayer coatings are compact, low-loss, and convenient to be integrated with other systems. Thus, they have been widely used in the field of dispersion enhancement and dispersion compensation, such as Chirped mirrors and Gires-Tournois mirrors^19^,^20^,^21^,^22^,^23^. In this paper, we demonstrate a new design of a broadband superluminal ring laser gyro by employing multilayer optical coatings with huge GD on one of the mirrors while keeping its high-reflectivity property. Meanwhile, we have computed the enhancement factor (*S_enh_*) of the superluminal gyro and examined a number of parameters including the FWHM bandwidth for *S_enh_*, the scale factor linearity, and the cavity length dependence for miniaturization issues. This GD involved superluminal RLG can greatly enhance the sensitivity of rotation measurement by a factor of 10^3^; while, the broadband FWHM can reach 20 MHz. On contrast to all its merits, this superluminal RLG is compact and beneficial for miniaturization.

## Results

**Figure 1:**
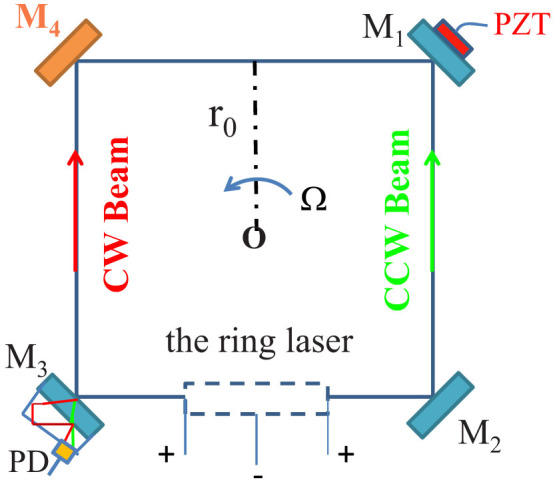
Schematic of superluminal ring laser gyro with four high-reflectivity mirrors (M_1_, M_2_, M_3_ and M_4_), in which M_4_ is the one with huge group delay induced by multilayer coatings, M_3_ is the output mirror. Lead zirconium titanate (PZT) plate on M_1_ is used to control the laser cavity length.

The transformation function for multilayer optical coatings can be written as: 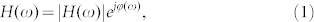
where |*H*(*ω*)| is the light amplitude, *ϕ*(*ω*) is the phase shift when light propagates through the multilayer coatings. *ϕ*(*ω*) can be expanded using Taylor series around *ω*_0_^22^: 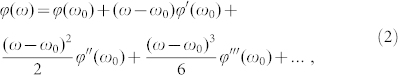
where 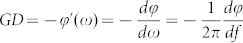 is defined as the group delay, 
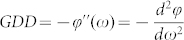
 is the group delay dispersion, *f* and *ω* are the frequency and angular frequency. Let us consider a square RLG with four high-reflectivity mirrors (M_1_, M_2_, M_3_ and M_4_), in which M_4_ is the one with a huge GD induced by multilayer coatings and M_3_ is the output mirror, as illustrated in Figure 1. The lead zirconium titanate (PZT) plate on M_1_ is used to control the laser cavity length. Compared to traditional RLGs, the only change is the multilayer coatings on M_4_, which requires minimal re-engineering for practical updating from old designs. Due to the huge GD induced by M_4_, Sagnac effects of the system need to be re-calculated as follows:

For an active RLG shown in Figure 1, the clockwise (CW) and counter-clockwise (CCW) ring laser modes have different frequencies (Δ*f* = *f*_−_ − *f*_+_) because of the difference in effective round-trip optical path lengths caused by the rotation of the cavity. Here, we denote *f*_±_, *λ*_±_ and 〈*L*_±_〉 as the frequencies, wavelengths and effective optical cavity lengths seen by the CW and CCW propagating beams, respectively. 〈*L*_±_〉 can be expressed as follows: 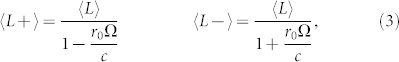
where 〈*L*〉 is the round-trip optical path length of the RLG without rotation, *r*_0_ is the radius of the beam path for ring cavities, Ω is the angular velocity rotating about the normal axis through the center of the interferometer, *c* is the speed of light. When considering the huge GD effects of M_4_, the frequency difference Δ*f* will induce additional phase difference. Thus, the resonance conditions for the CW and CCW propagating beams can be rewritten as: 
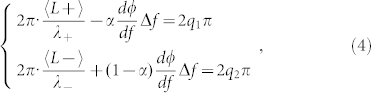
where *α* is a factor valued between 0 and 1, *q*_1_ and *q*_2_ are integers, *λ*_+_ = *c*/*f*_+_, *λ*_−_ = *c*/*f*_−_. Combining equation (3) and (4), we can obtain equation (5): 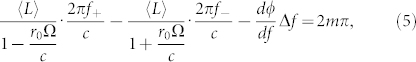
where *m* = *q*_1_ − *q*_2_. Assuming *m* = 0 and *r*_0_Ω ≪ *c*, we can express equation (5) using Taylor series and the first order terms can be expressed by: 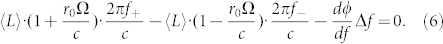


Under such approximation, we have *f*_+_ + *f*_−_ = 2 *f*_0_, where *f_0_* is the frequency of the CW and CCW beams in RLG without rotation. Then we can obtain the following result: 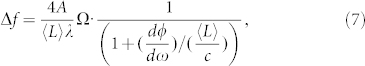
where 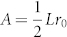 is the area enclosed by the beam path, *λ* is the wavelength of the CW and CCW beams at zero rotation. For 
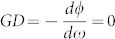
, 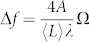, equation (7) becomes the formula for a usual RLG. For 
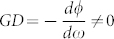
 (the case shown in our design), a sensitivity enhancement factor *S_enh_* for the superluminal RLG can be calculated as 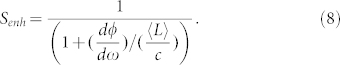


**Figure 2:**
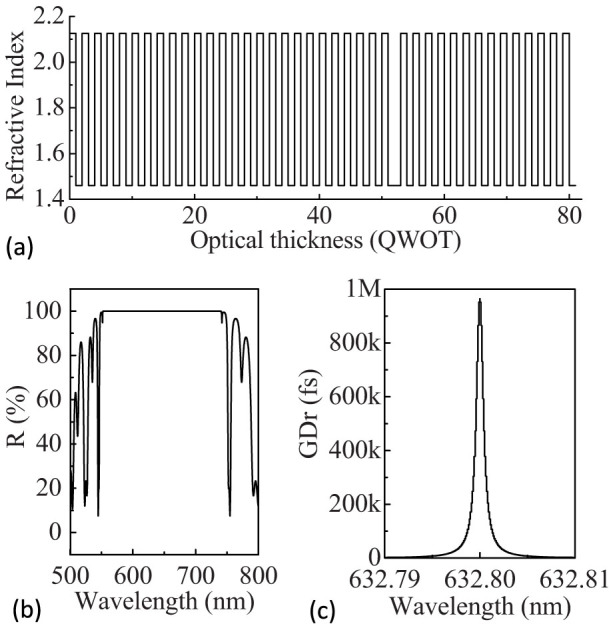
(a) Theoretical refractive-index profile of a high-reflectivity Ta_2_O_5_-SiO_2_ multilayer coatings (G/(HL)^25^H2L(HL)^14^/A) with huge group delay for M_4_. (b), (c) Computed reflectivity and group delay as a function of wavelength for the multilayer design of (a).

Therefore, when 
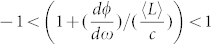
, |*S_enh_*| > 1, the scale factor of RLG is enlarged. For 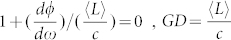, the enhancement factor reaches its maximum. Therefore, it is critical to design multilayer optical coatings with GD around 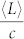, while maintaining the high-reflectivity property of the coatings for RLG. For multilayer optical coatings, there are many designs meeting above requirements. Considering practical applications, we choose the quarter-wave multilayer structure as follows: 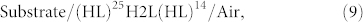
where H and L indicate quarter-wave optical layers with high and low refractive-index at 45° angle of incidence at a center wavelength of 632.8 nm. Figure 2(a) shows the theoretical refractive-index profile of a high-reflectivity Ta_2_O_5_-SiO_2_ multilayer coatings (G/(HL)^25^H2L(HL)^14^/A). In the case of ion beam sputtering (IBS) for the high-reflectivity mirrors, Ta_2_O_5_ is usually selected as high refractive-index material (n = 2.125), and SiO_2_ as low refractive-index material (n = 1.46). In fact, the multilayer structure of G/(HL)^25^H2L(HL)^14^/A is composed of a 23-layer high-reflectivity mirror ((HL)^11^H) and a 57-layer narrow bandpass filter ((LH)^14^2L(HL)^14^). Figure 2(b) and (c) show the computed reflectivity and GD curves as functions of wavelength for the RLG design illustrated in Figure 2(a). The calculated results suggest that this is a broadband high-reflectivity multilayer-coating system with huge GD (964228.61851 fs at maximum) at the central wavelength of 632.8 nm. At the maximum GD, for the cavity optical length of 289.55 mm, the enhancement factor *S_enh_* can reach as high as 1029 according to equation (8). Both the high-reflectivity mirrors in RLG and the bandpass filters in dense wavelength division multiplexing (DWDM) are widely used and the technology is mature, therefore this kind of superluminal RLG should be achievable.

**Figure 3:**
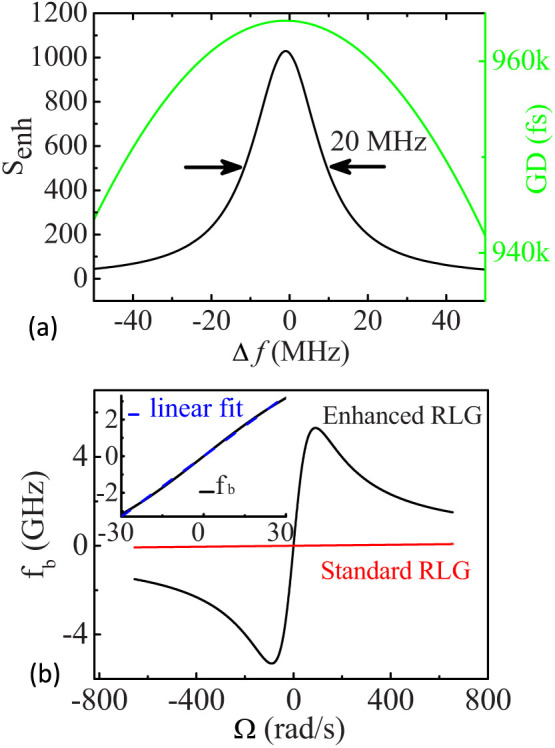
(a) Computed group delay (green curve) and sensitivity enhancement *S_enh_* (black curve) versus detuning of the laser frequency *Δf*, using the parameters provided in the text. The peak value of *S_enh_* ≈ 1029 occurs for Δ*f* = 0, and the FWHM bandwidth is ~20 MHz. (b) The beat frequency *f_b_* of both fast-light enhanced (black curve) and standard RLGs (red curve) with respect to the angular rotation rate Ω*_r_*. The inset shows an expanded view of the *f_b_* −Ω*_r_* curve for the fast-light enhanced RLG ranged from −30 rad/s to 30 rad/s. Its linear fitting is shown in dashed blue line.

Considering practical applications of the superluminal RLG, the FWHM bandwidths of GD and *S_enh_* are very important, which need to be larger than the linewidth of the laser cavity, otherwise extremely accurate control of cavity length is required^14^. Figure 3(a) shows the computed GD (in green) and *S_enh_* (in black) versus detuning of laser frequency *Δf* using parameters provided above. The peak value of *S_enh_* ≈ 1029 occurs at Δ*f* = 0 (*ω* = *ω*_0_ = 2*πc*/*λ*_0_, where *λ*_0_ = 632.8 *nm*), and the FWHM bandwidth of enhancement factor *S_enh_* is ~20 MHz. For a regular round trip loss *σ* ~ 300 ppm of the RLG, the linewidth of the ring laser cavity is 
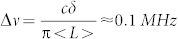
. Thus, the FWHM bandwidth of *S_enh_* of our design is two orders of magnitude higher than the linewidth of ring laser cavity. We have also calculated the beat frequency *f_b_* of fast-light enhanced RLG with respect to angular rotation rate Ω*_r_*. The relationship between the beat frequency of a standard RLG and the rotation rate is *f_b_* = 4*A*Ω*_r_*/(*L_r_λ*) according to equation (7), neglecting the effects of frequency lock-in. For a fast-light enhanced RLG, this expression needs to be modified to include the increased sensitivity term *S_enh_*: *f_b_* = 4*A*S*_enh_*Ω*_r_*/(*L_r_λ*). Figure 3(b) shows the beat frequency of both fast-light enhanced (black curve) and standard HeNe (red curve) RLGs with respect to the angular rotation rate. The inset gives an expanded view of the *f_b_* vs Ω*_r_* plot for fast-light enhanced RLG with a rotation rate ranged from −30 to 30 rad/s. It shows a perfect linear relation within this rotation speed range with its linear fitting shown in dashed blue line. This linear dynamic range of fast-light enhanced gyroscope is more than enough for usual navigation applications.

**Figure 4:**
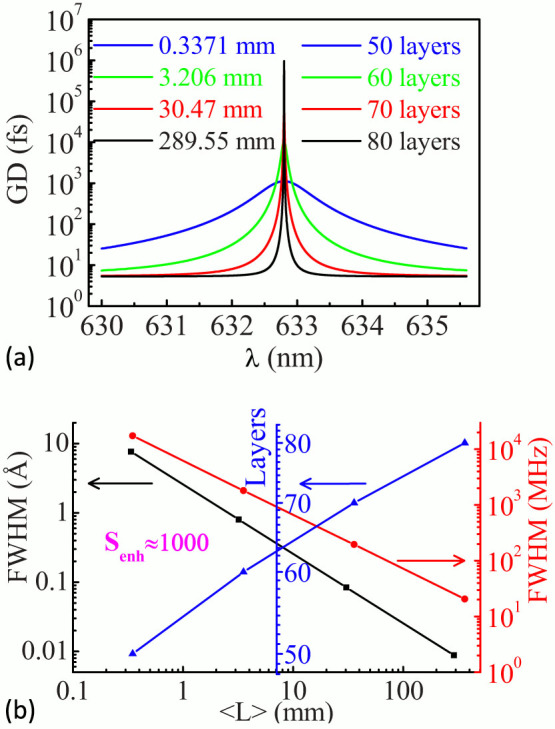
(a) Computed group delay as a function of wavelength for ring lasers with different cavity optical length <L> of 289.55 mm (black curve), 30.47 mm (red curve), 3.206 mm (blue curve) and 0.3371 mm (green curve), respectively. The corresponding multilayer coatings are 80 (G/(HL)^25^H2L(HL)^14^/A), 70 (G/(HL)^23^2H(LH)^11^L/A), 60 (G/(HL)^20^H2L(HL)^9^/A) and 50 layers (G/(HL)^18^2H(LH)^6^L/A), respectively. (b) The FWHM bandwidth of GD(λ) (in black), the FWHM bandwidth of *S_enh_* (Δ*f*) (in red) and the layers of multilayer coatings (in blue) as a function of laser cavity optical length <L>, respectively.

More importantly, equation (8) indicates that, in order to reach the same *S_enh_*, for shorter optical cavity length, the GD value required is smaller, which reduces the number of layers in the mirror coatings and greatly lowers the design and fabrication difficulty in applications. Figure 4(a) shows the calculated GD curves with respect to the wavelength for ring lasers at various optical cavity lengths of 289.55 mm (black line), 30.47 mm (red line), 3.206 mm (blue line), and 0.3371 mm (green line), respectively. The corresponding multilayer coatings are 80 (G/(HL)^25^H2L(HL)^14^/A), 70 (G/(HL)^23^2H(LH)^11^L/A), 60 (G/(HL)^20^H2L(HL)^9^/A) and 50 layers (G/(HL)^18^2H(LH)^6^L/A), respectively. This suggests that, when the optical cavity length <L> decreases, the required number of layers in the mirror coatings and their GD value decease. Meanwhile, the bandwidth of GD(λ) increases (Figure 4(a)), so that this type of fast-light enhanced RLG is beneficial for miniaturization. Figure 4(b) shows the FWHM bandwidth of GD(λ) (in black), the FWHM bandwidth of *S_enh_*(Δ*f*) (in red) and the layers of multilayer coatings (in blue) with respect to the laser optical cavity length <L>, respectively, where the maximum *S_enh_* is kept as constant at ~1000. It is obvious to see that, when the <L> decreases from 289.55 mm to 0.3371 mm, the number of layers required for the coatings deceases from 80 to 50; while, the GD(λ) FWHM bandwidth increases from ~0.01 Å to 7.64 Å. Therefore, the multilayer coatings with GD used for superluminal gyroscope can be realized more easily for RLGs with shorter optical cavity lengths. This multilayer optical coatings with a GD(λ) FWHM bandwidth of 7.64 Å can be easily realized by modern IBS method with optical monitoring of the central wavelength.

## Discussion

Compared with other fast-light media or systems for superluminal gyroscopes such as alkali metal vapor cells^7^,^8^, coupled optical resonators^9^, photorefractive crystals^10^, optical fibers^11^, spectral hole burning^12^ and rare atomic gasses^13^, the GD induced superluminal gyroscope has significant advantages as follows. Firstly, the multilayer-coating system with GD is based on the traditional RLG with updating of only one mirror, which requires minimal re-engineering and will not introduce additional backscattering. Through optimizing the design of multilayer optical-coating systems, the operating wavelength of this GD induced RLG can be tunable to meet different applications. Secondly, the FWHM bandwidth of the enhancement factor *S_enh_* is much larger than the linewidth of the ring laser cavity, which increases the tolerance of the system for deviations in cavity length. Thirdly, this type of fast-light enhanced RLG is beneficial for miniaturization. With the development of micro-cavity technology such as vertical cavity emission lasers (VCSEL)^24^,^25^,^26^ and integrated micro RLGs^27^,^28^,^29^,^30^, micro fast-light enhanced RLG is promising to be realized in future with the idea of using multilayer coatings with huge GD.

In summary, we have proposed and analyzed a superluminal ring laser gyro using multilayer optical coatings with huge GD. This type of superluminal RLG has a strong sensitivity enhancement and a broadband enhancement factor that requires minimal re-engineering, and advantageous for miniaturization. The idea of using GD coatings as fast-light media will shed lights on the design and application of fast-light sensors.

## Methods

Considering the GD of multilayer coatings, Sagnac effects of the superluminal RLG are calculated according to the principle of laser physics directly as shown in the text, which is simple, clear and accurate. The design and computation of multilayer coatings are done by OptiLayer Thin Film Software, two targets (reflectivity and GD) are set for evaluation.

## Author Contributions

T.L.Q. designed the superluminal gyroscope and performed the calculation. K.Y.Y. directed the research. X.H. carried out the calculation of Sagnac effects of the superluminal RLG when considering the GD of multilayer coatings. S.Y.W. assisted in the design and computation of multilayer coatings by Optilayer Software. T.L.Q. and K.Y.Y. prepared the manuscript and refined the paper. Y.H. and H.L. provided advices and helpful theoretical discussion. All authors discussed the results and contributed to the refinement of the paper.
